# Challenges of Congenital Heart Surgery in Brazil: It is Time to Designate Pediatric Congenital Heart Surgery Subspecialty

**DOI:** 10.21470/1678-9741-2024-0138

**Published:** 2024-05-16

**Authors:** Valdester Cavalcante Pinto Júnior, Leonardo Augusto Miana, Fábio Binhara Navarro, Bruno da Costa Rocha, Renato Samy Assad, Marcos Aurélio Barboza de Oliveira, Fábio Said Salum, Ulisses Alexandre Croti, Beatriz Helena Sanches Furlanetto, Marcelo Biscegli Jatene, Luiz Fernando Caneo, Andrey José de Oliveira Monteiro, Fernando Ribeiro de Moraes Neto, Fernando Antoniali, Pedro Rafael Salerno, Vinicius José da Silva Nina

**Affiliations:** 1 Department of Pediatric Cardiovascular Surgery, Instituto de Coração de Fortaleza, Fortaleza, Ceará, Brazil; 2 Department of Pediatric Cardiovascular Surgery, Hospital de Messejana, Fortaleza, Fortaleza, Ceará, Brazil; 3 Division of Cardiovascular Surgery, Instituto do Coração (InCor), Hospital das Clínicas da Faculdade de Medicina da Universidade de São Paulo, São Paulo, São Paulo, Brazil; 4 Department of Pediatric Cardiovascular Surgery, Hospital Pequeno Príncipe, Curitiba, Paraná, Brazil; 5 Department of Cardiovascular Surgery, Universidade Federal do Paraná, Hospital de Clínicas, Curitiba, Paraná, Brazil; 6 Department of Pediatric Cardiovascular Surgery, Hospital da Criança Martagão Gesteira, Salvador, Bahia, Brazil; 7 Departamento de Cirurgia Cardiovascular Pediátrica, Sociedade Brasileira de Cirurgia Cardiovascular, Brazil; 8 CardioPedBrasil® - Centro do Coração da Criança, Hospital da Criança e Maternidade de São José do Rio Preto - Fundação Faculdade Regional de Medicina/Faculdade de Medicina de São José do Rio Preto (FUNFARME/FAMERP), São José do Rio Preto, São Paulo, Brazil; 9 Hospital Beneficência Portuguesa de São Paulo, São Paulo, São Paulo, Brazil; 10 Hospital Infantil Sabará, São Paulo, São Paulo, São Paulo, Brazil; 11 Hospital das Clínicas, Faculdade de Medicina, Universidade de São Paulo, São Paulo, São Paulo, Brazil; 12 Pediatric Cardiac Surgery Unit, Cardiovascular Division, Instituto do Coração, Faculdade de Medicina da Universidade de São Paulo, São Paulo, São Paulo, Brazil; 13 Department of Cardiovascular Surgery, Universidade do Estado do Rio de Janeiro (UERJ), Rio de Janeiro, Rio de Janeiro, Brazil; 14 Instituto Pró-Criança, Rio de Janeiro, Rio de Janeiro, Brazil; 15 Instituto do Coração de Pernambuco, Recife, Pernambuco, Brasil; 16 Hospital Celso Pierro, Pontifícia Universidade Católica (PUC) de Campinas, Campinas, São Paulo, Brazil; 17 Division of Cardiovascular Surgery, Pronto-Socorro Cardiológico Universitário de Pernambuco Prof. Luiz Tavares (PROCAPE), Recife, Pernambuco, Brazil; 18 Hospital Universitário, Universidade Federal do Maranhão, São Luís, Maranhão, Brazil

**Keywords:** Congenital Heart Defects, Certification, Training, Education, Patient Care, Medical Societies

## Abstract

Congenital heart disease (CHD) affects eight to ten out of every 1,000 births, resulting in approximately 23,057 new cases in Brazil in 2022. About one in four children with CHD requires surgery or other procedures in the first year of life, and it is expected that approximately 81% of these children with CHD will survive until at least 35 years of age. Professionals choosing to specialize in CHD surgery face numerous challenges, not only related to mastering surgical techniques and the complexity of the diseases but also to the lack of recognition by medical societies as a separate subspecialty. Furthermore, families face difficulties when access to services capable of providing treatment for these children.

To address these challenges, it is essential to have specialized hospitals, qualified professionals, updated technologies, sustainable industry, appropriate financing, quality assessment systems, and knowledge generation. The path to excellence involves specialization across all involved parties.

As we reflect on the importance of Pediatric Cardiovascular Surgery and Congenital Heart Diseases establishing themselves as a subspecialty of Cardiovascular Surgery, it is essential to look beyond our borders to countries like the United States of America and United Kingdom, where this evolution is already a reality. This autonomy has led to significant advancements in research, education, and patient care outcomes, establishing a care model.

By following this path in Brazil, we not only align our practice with the highest international standards but also demonstrate our maturity and the ability to meet the specific needs of patients with CHD and those with acquired childhood heart disease.

**Table:** 

Abbreviations, Acronyms & Symbols				
AMB	= Associação Médica Brasileira		SBCCV	= Sociedade Brasileira de Cirurgia Cardiovascular
CHD	= Congenital heart disease		SUS	= Sistema Único de Saúde
DATASUS	= Departamento de Informática do SUS		UK	= United Kingdom
DCCVPed	= Departamento de Cirurgia Cardiovascular Pediátrica		USA	= United States of America

*“Understanding is the beginning of approval.” Baruch Espinoza*

In the Brazilian state, the number of births has steadily decreased over the past decades. In 2002, the number of registrations in the Sistema de Recuperação Automática (or SIDRA) of the Instituto Brasileiro de Geografia e Estatística (or IBGE) was 3,865,419^[1]^, unlike in 2022, when the total recorded was 2,561,922^[2]^, a decrease of 30% in birth registrations over 20 years. Congenital heart disease (CHD) occurs in eight to ten out of every 1,000 births. Therefore, in 2022, there were approximately 23,057 cases of CHD.

About one in four children with CHD presents in a critical condition and generally requires surgery or other procedures in the first year of life^[3]^. It is expected that around 81% of infants born with CHD, critical or not, will survive until at least 35 years of age^[4]^. About 30% of patients with CHD will require multiple surgeries for physiological and anatomical reasons, undergoing repairs in stages. Another 15% will require reoperations for conduit replacements, valve repairs/replacements^[5]^. Additionally, reinterventions for palliative procedures are more frequent in the treatment process of critical CHD. Therefore, reoperations for CHD are common, potentially accounting for up to one-third of all operations, and almost all patients will require some form of lifelong care^[6]^.

In this report, we have confined our analysis to CHD, but we acknowledge the existence of children with cardiac tumors, Marfan syndrome, cardiomyopathy, primary arrhythmia, rheumatic heart disease, and various other cardiac problems that also require diagnosis, treatment, and follow-up. Thus, we underestimate the number of patients comprising the field of Pediatric Cardiology and Cardiovascular Surgery and Congenital Heart Diseases.

Indeed, this is the universe of CHD, from fetus to adult, which must be cared for in the dimensions of diagnosis (clinical and imaging), treatment (surgical and/or interventional or clinical), and post-treatment follow-up.

Therefore, CHD has a significant aspect of public health and is a lifelong commitment^[5]^, which prompts the demand for understanding the limiting factors to greater access to treatment.

What is intended for minorities and those with specific needs (here considering the cardiac child as such) stems from the struggles of individuals, family members, and professionals shocked by the negligence of those who, by obligation, should be responsible for the effectiveness of health policies. Public policies are largely formulated to respond to a larger contingent. Yet, believe it: those individuals exist!

The specific needs of this population require investments in technologies that demand continuous innovation and diversification, which increase production costs. In addition, limited demand based on the lower expected surgical volume as compared to adult acquired heart diseases make these inputs expensive, resulting in low return on investment. This is surely the reason why the industry is not prioritizing these groups when developing new products^[7]^.

We invoke a premise of health policies, extolled in political speeches but poorly implemented in practice - equity (to ensure everyone has the same chance to access to health and to live healthy). With this intention, we bring to light the Constituição da República Federativa do Brasil (Article 196)^[8]^.

“Health is the right of all and the duty of the State to ensure …universal and equal access to actions and services for its promotion, protection, and recovery.”

As the individuals in question are children or their condition originates in childhood, it is worth also quoting the Estatuto da Criança e do Adolescente (or ECA) Law No. 8,069, of July 13, 1990^[9]^.

“Article 4. It is the duty …of the public authorities to ensure, with absolute priority, the realization of rights related to life, health...”

To follow on these mandates and undertake responsible and citizen-centered care for these children, hospital institutions must, through norms and healthcare needs, offer updated infrastructure and qualified professionals - a multidisciplinary team - with a proper understanding of the administration of interdependent clinical processes. Overcoming this challenge should be the norm, but in daily practice, institutions either give up or work below targets, often due to low reimbursement for services contracted by the Sistema Único de Saúde (SUS).

According to data analysis from the Departamento de Informática do SUS (DATASUS), there is a deficit in the care for cardiac children that exceeds 80% in some states of the federation^[10]^. There is no shortage of norms or public policies for the sector; these exist and are reissued, but along with promises that, to the discerning eye, are nothing more than diversions. Investments commensurate with the complexity of the children under consideration are scarce^[11]^.

It is crucial that we understand this care environment is supported by specialized hospitals, qualified professionals, updated technologies, sustainable industry, appropriate financial and administrative management, quality assessment systems, and knowledge generation and transfer.

There is no official database for the analysis of CHD distributed by risk stratification. The large national registry, DATASUS, offers general data on births, hospitalizations, and mortality, allowing for general analyses such as the incidence of CHD and estimates of diagnostic deficits^[12]^. While comprehensive, DATASUS is insufficient to address the needs of the CHD population. For detailed interpretation, some centers organize their own databases or through international partnerships, seeking to evaluate results, improve the quality of local care, and generate teaching and research^[7,12-15]^.

In our country, we witness the neglect in implementing these premises, as we experience situations such as healthcare gaps in certain regions, lack of infrastructure, and insufficient qualified professionals, exposing the reality of inadequate access and treatment. All this creates a feeling of frustration for the specialists who have dedicated their lives to help individuals with CHD.

In the rest of this manuscript, we would like to focus on the challenges and opportunities in training pediatric cardiovascular surgeons. This is not meant to neglect the need for specific training and continuous education of all other specialties and professions involved in pediatric cardiac care. The evaluation of the other pillars of care for CHD will be the subject of other publications.

It is encouraging that we call the professionals who operate on cardiac children by the appropriate name, which, as an area of expertise in Cardiovascular Surgery dedicated to Pediatrics is now widely accepted. This understanding is straightforward, and facts support it: the Departamento de Cirurgia Cardiovascular Pediátrica (DCCVPed) of the Sociedade Brasileira de Cirurgia Cardiovascular (SBCCV) was founded 21 years ago; the standards for the accreditation of Pediatric Cardiovascular Surgery Units, alongside the Ministry of Health (MS) exist since 2004; and there are several teaching centers with training programs in Pediatric Cardiovascular Surgery in many Brazilian institutions (Instituto do Coração, Instituto Dante Pazzanese, Beneficência Portuguesa, Hospital do Coração - São Paulo/São Paulo; Hospital da Criança e Maternidade - São José do Rio Preto/São Paulo; Hospital Pequeno Príncipe - Curitiba/Paraná; Hospital da Criança Martagão Gesteira - Salvador/Bahia; Santa Casa de Porto Alegre - Porto Alegre/Rio Grande do Sul).

We invite the reader’s reflection on the events described in the Figure 1, each being a reaction to legitimate provocations and becoming a demand for further action.

**Fig. 1:**
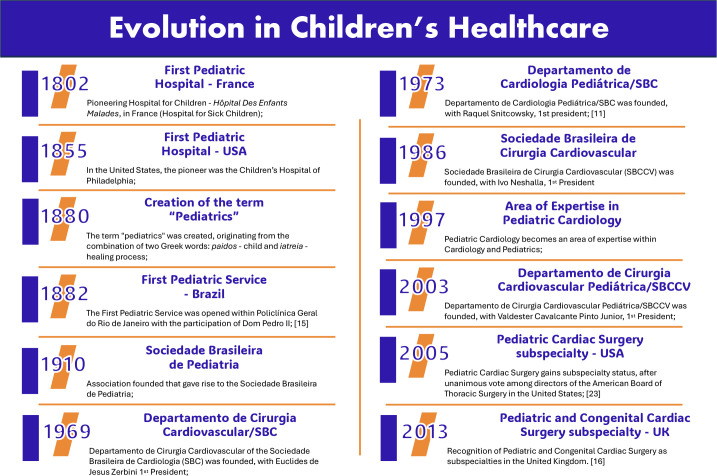
Evolution in children’s healthcare. UK=United Kingdom; USA=United States of America.

Under current conditions in Brazil, we cannot use the term Pediatric Cardiovascular Surgeon, as the area of expertise is still in the process of approval by SBCCV, and consequently, by the Associação Médica Brasileira (AMB) and of registration by the Conselho Federal de Medicina (or CFM). Consequently, certification in the field by SBCCV/DCCPed/AMB, public competitions for the admission of these professionals, the creation of unified training programs, and remuneration during training through scholarships from the Ministry of Education are impossible.

Pediatric Cardiovascular Surgery and Congenital Heart Disease have traversed a long road in a short period, originating from thoracic surgery about 60 years ago. Subspecialization undoubtedly contributed to the current landscape of excellent outcomes in various parts of the world.

The subspecialty was recognized as a separate discipline in 2005 in the United States of America and in 2013 in the United Kingdom of England, Scotland, Wales, and Northern Ireland, providing an opportunity for the development of specific skills for residents, such as work in the surgical laboratory, for example. This established the prerequisites for extracorporeal circulation and surgical technique, as well as immersion in pathophysiology, with anatomical heart models (*in vivo* or derived from 3D printing), aiming at the personal growth of the future surgeon and collaborative work with the multidisciplinary team (clinical pediatric cardiologists, psychologists, physiotherapists, nurses, social workers, and others). In this way, it maximized the understanding of the impact of CHD on the individual, the family, and the society receiving care^[13,16,17]^.

Other countries in Europe, such as Bulgaria, France, Ireland, the Netherlands, Poland, and Romania, also have national training programs in congenital cardiology. Ireland, the Netherlands, Spain, and England already certify surgeons graduating from training centers as congenital surgeons^[18]^.

It is crucial to highlight the role of the DCCVPed/SBCCV over 21 years ago. The seminal purpose of its establishment was to ensure the representation of associates dedicated to the pediatric area, before the Ministry of Health, aiming at reducing inequalities in the care of children with heart diseases. As a consequence, the Ministry of Health published Ordinance No. 210 in 2004^[19]^, under which Pediatric Cardiovascular Surgery was recognized when creating Pediatric Cardiovascular Surgery Units. This qualification already enables institutions to perform surgeries on heart disease patients under 18 years of age. Today, we have 54 accredited centers in Brazil. This initial action was followed by numerous representations, always articulated with SBCCV, such as participation in the drafting of Ordinance No. 1,727 of July 11, 2017^[20]^, which approved the Plano Nacional de Assistência à Criança com Cardiopatia Congênita, and in the project for situational diagnosis of surgery deficits in Brazil, carried out in partnership with the Ministry of Health, Programa de Apoio ao Desenvolvimento Institucional do SUS (or Proadi) - Ministry of Health. In the educational environment, there is an untiring disposition to hold symposia and congresses, continuous publications, as well as to demand on-site solutions for services^[7,21]^. One such example is the publication of the book “Cardiologia e Cirurgia Cardiovascular Pediátrica” (Ulisses Croti, Valdester Pinto Jr, Sandra Mattos, Vera Aielo, and Valéria Moreira), 1^st^ edition - 2008^[22]^ and 2^nd^ edition - 2013, which contributes to the training of surgeons, clinicians, and related professionals.

The DCCVPed/SBCCV counted on the commitment of ten boards since its inception. Their presidents were (in chronological order): Valdester Cavalcante Pinto Júnior, Fábio Said Salum, Ulisses Alexandre Croti, Beatriz Helena Sanches Furlanetto, Marcelo Biscegli Jatene, Luiz Fernando Caneo, Fernando Ribeiro de Moraes Neto, Andrey José de Oliveira Monteiro, Fernando Antoniali, Pedro Rafael Salerno, and Leonardo Augusto Miana. We would like to specifically acknowledge the determination and commitment of Dr. Jarbas Jakson Dinkhuysen, president of SBCCV in the creation and approval of DCCVPed.

Throughout its 50 years, SBCCV has been tireless in defending the right to treatment for children, having, on several occasions, achieved improvements in medical remuneration, reimbursement for hospital procedures, and financial reimbursement for specific surgical supplies. We would like to honor and express our gratitude to all those who have dedicated and continue to dedicate their lives to this cause, notably DCCVPed.

When reflecting on the importance of Pediatric Cardiovascular Surgery and Congenital Heart Diseases establishing itself as a subspecialty of Cardiovascular Surgery, it is essential to look beyond our borders, to the experiences of countries like the United States of America and England, where this evolution is already a reality. This autonomy has led to significant advances in research, teaching, and patient care outcomes, establishing a care model that is both aspirational and inspiring for the whole world^[16,23]^.

In Brazil, achieving this subspecialty status, will not only align our practice with the highest international standards but also demonstrate our maturity and ability to meet the specific needs of patients with CHD and those with acquired heart disease in childhood.
